# Differential signaling mechanism for HIV-1 Nef-mediated production of IL-6 and IL-8 in human astrocytes

**DOI:** 10.1038/srep09867

**Published:** 2015-06-15

**Authors:** Xun Liu, Anil Kumar

**Affiliations:** 1 Division of Pharmacology and Toxicology, School of Pharmacy, University of Missouri, Kansas City, MO 64108

## Abstract

Variety of HIV-1 viral proteins including HIV-1 Nef are known to activate astrocytes and microglia in the brain and cause the release of pro-inflammatory cytokines, which is thought to be one of the mechanisms leading to HIV-1- mediated neurotoxicity. IL-6 and IL-8 have been found in the CSF of patients with HIV-1 associated dementia (HAD), suggesting that they might play important roles in HIV-1 neuropathology. In the present study we examined the effects of HIV-1 Nef on IL-6 and IL-8 induction in astrocytes. The results demonstrate that both IL-6 and IL-8 are significantly induced in HIV-1 Nef-transfected SVGA astrocytes and HIV-1 Nef-treated primary fetal astrocytes. We also determined the molecular mechanisms responsible for the HIV-1 Nef-induced increased IL-6 and IL-8 by using chemical inhibitors and siRNAs against PI3K/Akt/PKC, p38 MAPK, NF-κB, CEBP and AP-1. Our results clearly demonstrate that the PI3K/PKC, p38 MAPK, NF-κB and AP-1 pathways are involved in HIV-1 Nef-induced IL-6 production in astrocytes, while PI3K/PKC and NF-κB pathways are involved in HIV-1 Nef-induced IL-8 production. These results offer new potential targets to develop therapeutic strategy for treatment of HIV-1 associated neurological disorders, prevalent in > 40% of individuals infected with HIV-1.

The introduction of highly active antiretroviral therapy (HAART) has resulted in a decrease in the prevalence of HIV-1 associated dementia (HAD) and overall mortality in HIV-1 infected patients^1^. However, a significant proportion of these patients suffer from the milder form of HIV-associated neurocognitive disorders known as minor cognitive motor disorders (MCMD)^2^. HIV enters the CNS via a “Trojan Horse” mechanism, which involves the infiltration of infected monocytes across BBB and activation of microglia and macrophages in the brain^3^. Those activated cells then produce viral proteins, which can result in direct neurotoxicity. These viral proteins can also activate uninfected cells, causing indirect neurotoxicity by the secretion of toxic mediators such as arachidonic acid metabolites, as well as pro-inflammatory cytokines/chemokines^4^.

Astrocytes, the most abundant cell type in the CNS, have numerous functions in brain physiology, including neuronal migration, maintenance of BBB integrity, and modulation of immune responses^5^. Furthermore, astrocytes play an important role in HIV-1-mediated neuropathology, in that they secrete inflammatory mediators and serve as a viral reservoir. It has been reported that nearly 20% of astrocytes carry HIV-1 DNA in brain tissues obtained from HIV-1 infected individuals^6^,^7^. Although astrocytes were previously considered to be subject to a low level of productive infection with HIV-1, in a recent study human fetal astrocytes showed persistant infection even up to 160 days after HIV-1 pseudovirus infection^8^.

HIV-1 Nef is a multifunctional viral accessory protein of 27–35 kd, and it is abundantly expressed before integration of HIV-1^9^. Notably, expression of the HIV-1 Nef gene alone in CD4+ T cells and macrophages was sufficient to induce an AIDS-like phenotype in transgenic mice, resulting in symptoms of immunodeficiency and depletion of CD4+ cells^10^,^11^. Although the functions of HIV-1 Nef in the periphery have been well established in HIV-1 infection, fewer studies have focused on the effects of HIV-1 Nef in the CNS. Nevertheless, HIV-1 Nef mRNA and protein has been shown to be present in brain cells, specifically astrocytes of individuals with AIDS-associated neuropathology^12^,^13^. HIV-1 Nef has been demonstrated to be toxic to human neurons *in vitro*, and to cause the release of soluble factors such as CCL2, IL-6, TNF-α and IFN-γ when expressed in astrocytes^14^,^15^,^16^. In addition, the neuroinflammation and cytotoxicity induced by HIV-1 Nef is often associated with behavioral changes. One research group has transplanted HIV-1 Nef-transduced macrophages into the hippocampus of rats and shown increased recruitment of monocytes/macrophages into the CNS as well as cognitive changes^17^. In another study, impairment of spatial and recognition memory was seen along with an increase of CCL2 secretion after implantation of the HIV-1 Nef-transfected astrocytes into rat hippocampus^18^.

IL-6 is a 26-kDa proinflammatory cytokine produced by a variety of cells. It is an activator of acute phase responses and the overproduction of IL-6 was seen in a variety of chronic autoimmune and inflammatory diseases, including rheumatoid arthritis (RA) and inflammatory bowel disease^19^. Moreover, Studies have demonstrated that high levels of IL-6 may serve as a biomarker both for activation-induced CD4+ T-cell losses in patients with advanced HIV-1 infection as well as for increased mortality in HIV-1 infected individuals^20^,^21^. The importance of IL-6 in neuroinflammation and HAND was indicated in a few studies, in which elevated levels of IL-6 were found in the CSF of patients with AIDS dementia complex and with milder forms of HAND^22^,^23^,^24^.

IL-8/CXCL8, a member of CXC chemokine sub-family, was originally identified as a neutrophil-activating chemokine, but it also plays a role in chemotaxis and activation of monocytes and T cells^25^,^26^,^27^. IL-8 can work synergistically with CCL2 to mediate monocyte migration to sites of inflammation^28^. A recent study reported that the IL-8 level in the CSF in HAD patients was significantly higher than in HIV-1 seropositive individuals without neurological complications^29^. Furthermore, one research group has demonstrated that IL-8 inhibits long-term potentiation via CXCR2 receptor in the CA1 region of rat hippocampus, suggesting that IL-8 might play a role in neuronal dysfunction^30^.

Taken together, both IL-6 and IL-8 play important roles in neurotoxicity caused by HIV-1. However, the mechanism(s) of IL-6 and IL-8 production by HIV-1 and/or its viral proteins are still under investigation. In the present study we investigated the role of HIV-1 Nef in the induction of IL-6 and IL-8 in human astrocytes. Then, the mechanisms responsible for the HIV-1 Nef-mediated increase of IL-6 and/or IL-8 were determined, particularly the involvement of NF-κB, AP-1, C/EBP, PI3K-Akt/PKC and p38 MAPK pathways.

## Results

### HIV-1 Nef treatment induces IL-6 and IL-8 production in SVGA cells and primary human fetal astrocytes (HFA)

**Figure 1:**
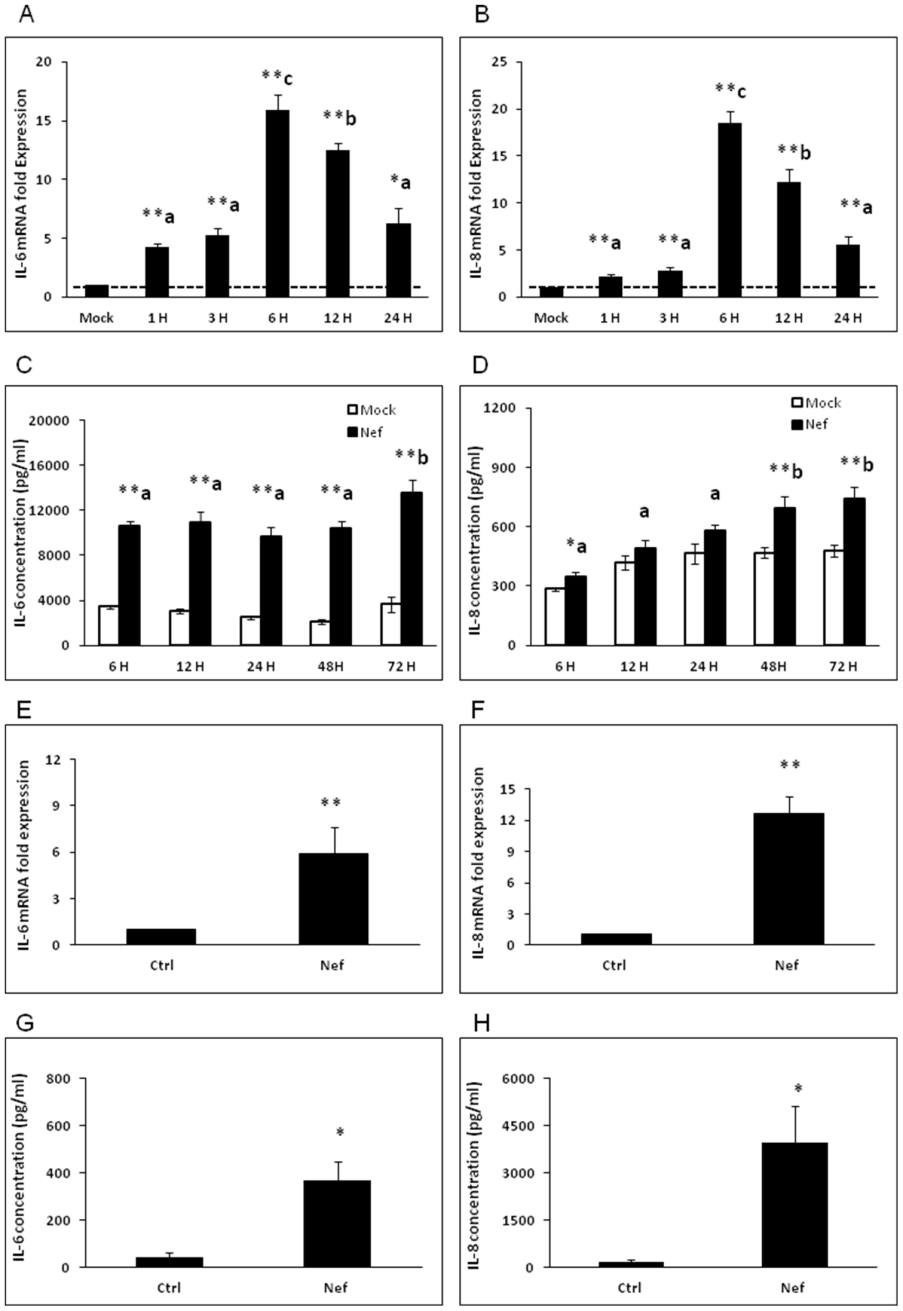
HIV-1 Nef induces IL-6 and IL-8 in SVGA astrocytes and human primary fetal astrocytes. 7 × 10^5^ SVGA astrocytes were seeded in a 6-well plate and were transfected with a plasmid encoding HIV-1 Nef for 5 h using Lipofectamine 2000™. The cells were harvested at the indicated times and RNA was isolated and the expression levels of IL-6 and IL-8 mRNA were determined by real time RT-PCR. Data in figures show fold-change relative to the mock-transfected wells at each time point (A–B). IL-6 and IL-8 concentrations released in the supernatants were measured at times indicated in the figure by multiplex cytokine assay (C–D). Each of the bars represents the mean ± SE of three independent experiments which were performed in triplicate. 1 × 10^6^ SVGA astrocytes were seeded in 12-well plates and were treated with HIV-1 Nef. The cells were harvested at 1 h ~ 6 h for total RNA isolation and the expression levels of IL-6 and IL-8 mRNA were determined by real time RT-PCR. Data in figures show the mean of peak expression of IL-6 and IL-8 depicted by fold-change relative to the untreated wells for all 6 donors (E–F). In separate experiments, IL-6 and IL-8 concentrations released in the supernatants were measured in 2 of 6 donors at 24 h by multiplex cytokine assay and data in figures show the mean of IL-6 and IL-8 concentrations (G–H). Each of the bars represents mean ± SE of individual donors. Statistical analyses were performed using one-way ANOVA. * represents p-value ≤ 0.05 and ** represents p-value ≤ 0.01. An LSD post-hoc test was performed to compare cytokine expression levels at different time points. Treatments that are significantly different from each other (i.e. p ≤ 0.05) are labeled with different letters.

We first wanted to determine whether human astrocytes produced IL-6 and IL-8 when stimulated by HIV-1 Nef. SVGA cells, an immortalized astrocytic cell line, were transfected with a plasmid encoding HIV-1 Nef and cells were harvested and IL-6 and IL-8 was measured at the level of mRNA. Supernatants were collected at the various time points and protein levels of these cytokines were determined. At 5 time points from 1 h to 24 h after transfection, the mRNA levels of both IL-6 and IL-8 were significantly higher in HIV-1 Nef-transfected cells than in the mock-transfected controls. The mRNA levels of IL-6 and IL-8 both peaked at 6 h post-transfection, at which time the HIV-1 Nef-transfected cells expressed IL-6 and IL-8 at levels that were 15.9 ± 1.3 fold and 18.5 ± 1.3 fold higher than that in mock-transfected controls, respectively. (Fig. 1A–B). IL-6 and IL-8 protein levels were determined using multiplex cytokine assay. IL-6 protein was found to be elevated after transfection with HIV-1 Nef for at least 72 hours post-transfection, at which time the concentration of IL-6 in the supernatant of HIV-1 Nef-transfected cells was 13.53 ± 1.17 ng/ml (Fig. 1C). The IL-8 levels from the supernatants of HIV-1 nef-transfected cells were significantly higher than those in mock-transfected cells starting at 6, 48 and 72 h post-transfection (Fig. 1D).

Next, we wanted to confirm that HIV-1 Nef could also induce IL-6 and IL-8 expression in human fetal astrocytes (HFA). We obtained HFA from 6 different donors and the cells were treated with 20 nM HIV-1 Nef recombinant protein. After a series of time points ranging from 1 h to 6 h, the cells were harvested for total RNA isolation which was then analyzed by real-time RT-PCR, and cell culture supernatants were collected at 24 h and analyzed using a multiplex cytokine assay. Significant up-regulation of IL-6 mRNA was seen in 5 out of 6 donors when treated with HIV-1 Nef. For donors 5 and 6, which showed higher expression levels of IL-6 mRNA, higher levels of IL-6 protein were also found in HIV-1 Nef-treated cells compared with untreated controls (see Supplementary Fig. S1). Similarly, significant up-regulation of IL-8 mRNA was seen in all 6 donors when treated with HIV-1 Nef. For donors 5 and 6, which showed relatively higher fold expression levels of IL-8 mRNA, IL-8 protein concentrations were analyzed and the results showed a higher level of IL-8 protein in HIV-1 Nef-treated cells compared with untreated controls (see Supplementary Fig. S2).The mean of IL-6 and IL-8 peak expression levels of all donors were 5.9 ± 1.7 fold and 12.7 ± 2.0 fold higher than that in untreated controls at the level of mRNA, respectively (Fig 1E–F). The mean of IL-6 and IL-8 protein expression levels reached 370.4 ± 78.0 pg/ml and 3946.9 ± 1189.6 pg/ml, respectively (Fig. 1G–H), both significantly higher than that in untreated controls.

### HIV-1 Nef-mediated induction of IL-6 and IL-8 in SVGA cells by confocal microscopy

**Figure 2:**
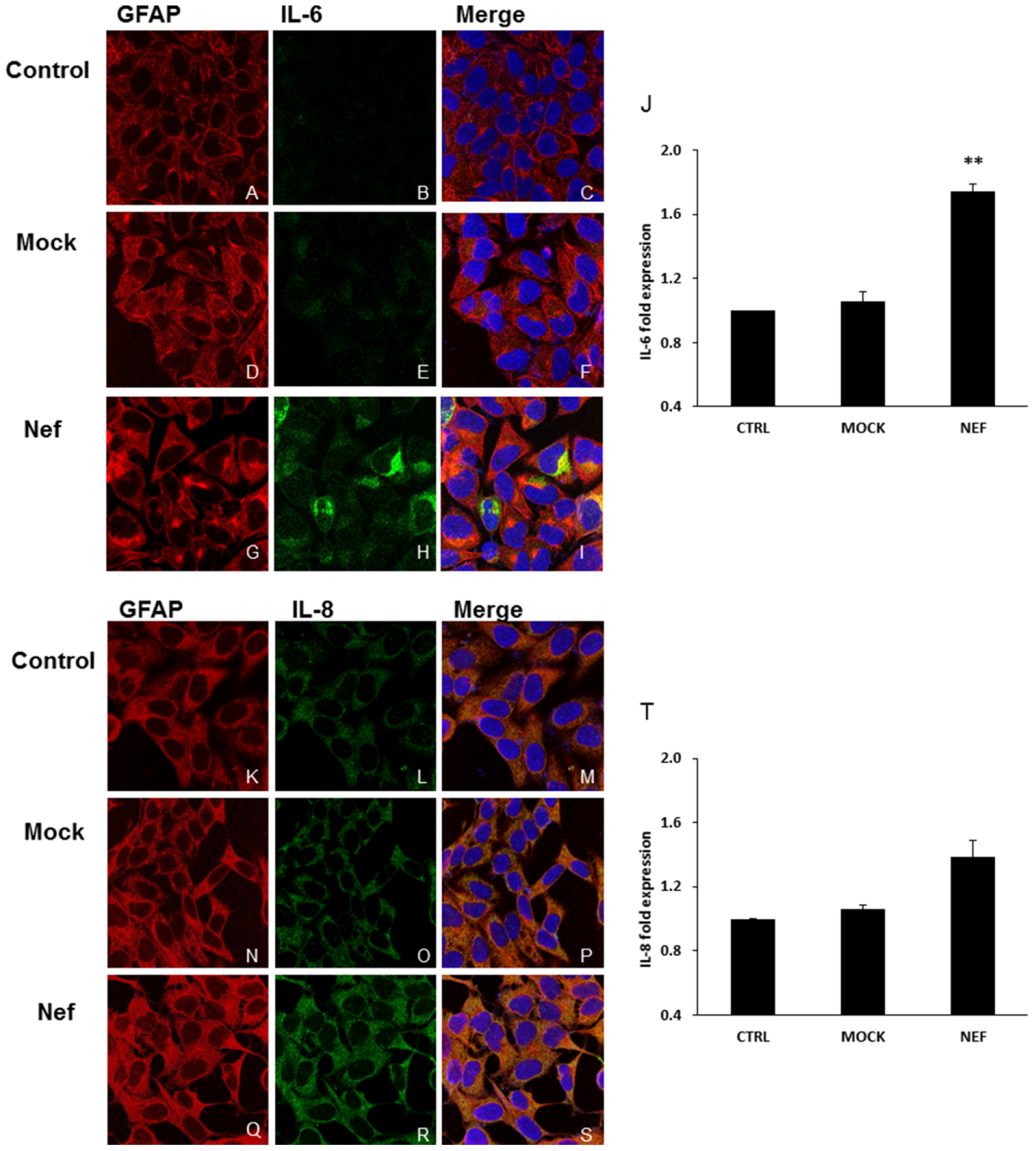
Confocal microscopy of IL-6 and IL-8 induced by HIV-1 Nef in astrocytes. 7 × 10^5^ SVGA astrocytes were seeded on a cover slip in 6-well plates and were either mock-transfected (D–F and N–P) or transfected with a plasmid encoding HIV-1 Nef (G–I and Q–S) for 5 h using Lipofectamine 2000™. Untreated controls (A–C and K–M) were used to visualize basal expression of IL-6 and IL-8 in astrocytes, respectively. 6 h later, cells were incubated with primary antibodies against CCL5 and GFAP and appropriate secondary antibodies labeled with Alexafluor 488 (IL6/IL-8) and Alexafluor 555 (GFAP). As illustrated in the figure, cells were stained for DAPI (blue); IL-6/IL-8 (green) and GFAP (red) and the images were captured using a Leica TCS SP5 II fluorescent microscope with 40× zoom oil emersion lens. The fold expression of IL-6 and IL-8 was calculated relative to GFAP and normalized with controls (J and T). Image analysis was performed using ImageJ software. Each of the bars represents mean ± SE of 3 different fields. Statistical analysis was performed using one-way ANOVA with an LSD post-hoc test in which * represents p-value ≤ 0.05 and ** represents p-value ≤ 0.01.

To confirm our findings regarding IL-6 and IL-8 induction by HIV-1 Nef, SVGA cells were transfected with a plasmid encoding HIV-1 Nef, and mock-transfections were also performed. At 6 h post-transfection cells were fixed and immunostained with a cocktail of antibodies against GFAP and IL-6/IL-8 and then stained with appropriate secondary antibodies. Fluorescence was detected by confocal microscopy and DAPI staining was used to visualize the nuclei. The levels of IL-6 and IL-8 (shown in green) were much higher in HIV-1 Nef-transfected cells than those in mock-transfected cells or untreated controls (Fig. 2A–I, Fig. 2K–S, respectively). Further, the mean of IL-6 expression levels of three different fields selected was significantly higher (~ 1.74 fold) than that in untreated controls (Fig. 2J). The mean of IL-8 expression levels was ~ 1.39 fold higher than that in untreated controls (Fig. 2T).

### HIV-1 Nef induces NF-κB p65 translocation as well as IκBα and IKK phosphorylation

**Figure 3:**
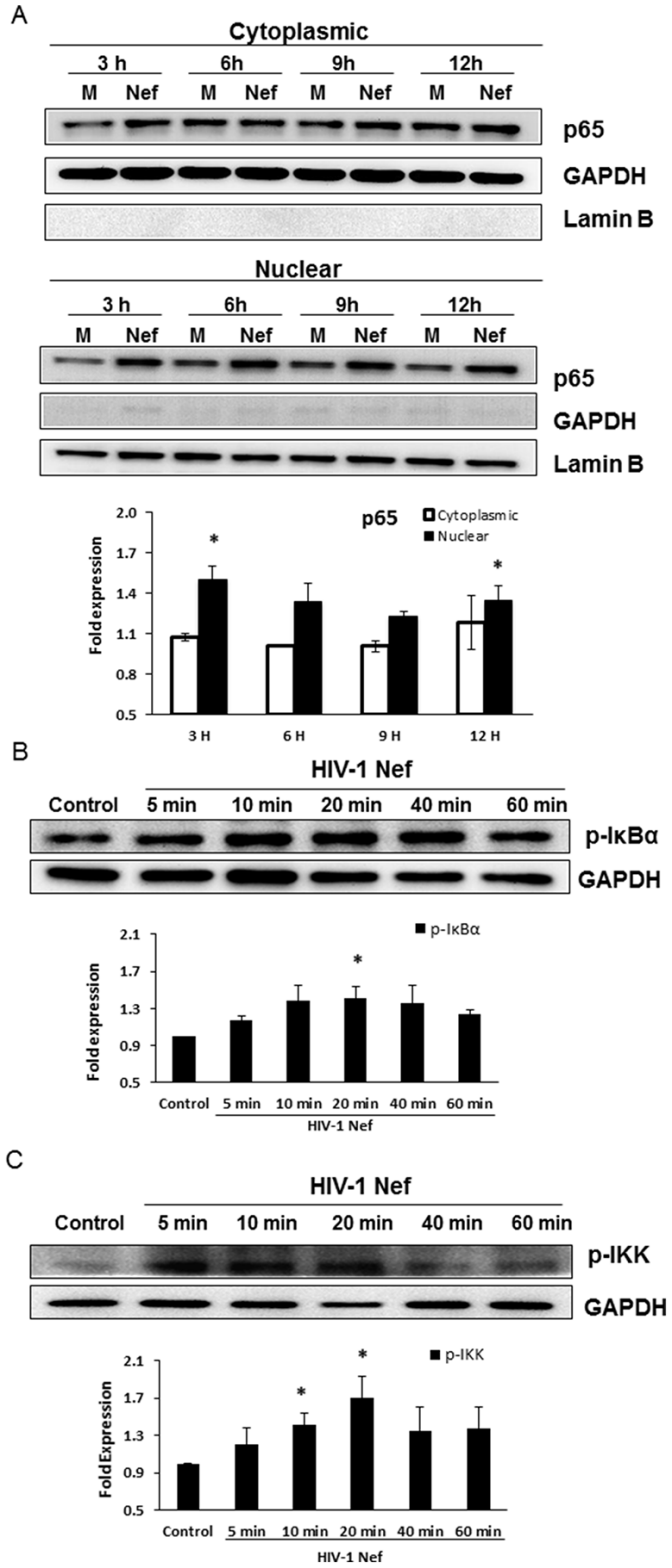
HIV-1 Nef induces NF-κB p65 translocation as well as the phosphorylation of IκBα and IKK. 7 × 10^5^ SVGA astrocytes were seeded in 6-well plates and were transfected with a plasmid encoding HIV-1 Nef using Lipofectamine 2000™. The transfection start time was considered time 0. The cells were harvested at the indicated times for isolation of cytoplasmic and nuclear proteins. The expression levels of p65 were determined in the cytosol and nucleus by western blotting. The figure shows one set of data that is representative of 3 independent experiments. Levels of p65 were quantified by spot densitometry and are presented as mean ± SE of 3 independent experiments. The fold expression of p65 was calculated relative to that of the mock-transfected wells at each individual time point (A). GAPDH was used as a loading control for cytoplasmic extracts and Lamin B was used as a loading control for nuclear extracts. In separate experiments, astrocytes were seeded in 6-well plates and were treated with recombinant HIV-1 Nef at the concentration of 20 nM. The cells were harvested at the indicated times for preparation of whole cell lysates followed by western blotting for determination of p-IκBα and p-IKK levels. The figure shows one set of data that is representative of 3 independent experiments. Levels of phosphorylated-proteins were quantified by spot densitometry and are presented as mean ± SE fold *vs*. control of 3 independent experiments (B–C). GAPDH was used as a loading control. Statistical analysis was performed using one-way ANOVA with an LSD post-hoc test in which * represents p-value ≤ 0.05 and ** represents p-value ≤ 0.01.

NF-κB dimers, most often the p65/p50 heterodimers, bind to the inhibitory IκBα in the cytosol. The dimers can be translocated into the nucleus upon phosphorylation and dissociation of IκBα, which is regulated by the IκB kinase (IKK) complex comprised of three subunits IKK1 (IKKα), IKK2 (IKKβ) and IKKγ. So we first sought to determine whether exposure of astrocytes to HIV-1 Nef results in p65 activation and translocation into the nucleus. Cells were transfected with the HIV-1 Nef-expressing plasmid for different periods of time and then nuclear and cytoplasmic protein fractions were isolated and assayed for p65 expression by western blot analysis. As expected, there was a significant increase in nuclear localization (or translocation) of p65 in HIV-1 Nef-transfected cells compared with that of mock-transfected controls. The maximum increase was seen at 3 h, and the level of p65 in the nuclear fraction in HIV-1 Nef-transfected cells was 1.5 fold higher than that in mock-transfected cells (Fig. 3A).

We then wanted to determine whether HIV-1 Nef causes the phosphorylation of IκBα and/or IKK. The levels of phosphorylation of IκBα and IKK1/2 in whole cell lysates of SVGA treated with HIV-1 recombinant Nef protein were measured by western blot (Fig. 3B–C). Although the peak level was observed at different time points in each individual experiment, there was a trend of increased phosphorylation of IκBα and the level of p-IκBα in HIV-1 Nef-treated cells was significantly higher than that in untreated controls at 20 min after HIV-1 Nef exposure (Fig. 3B). Similarly, there was time-dependent increase in p-IKK until 20 min after HIV-1 Nef treatment at which time point a 70% increase of p-IKK was seen in HIV-1 Nef-treated cells compared with untreated controls (Fig. 3C).

### NF-κB pathway is involved in HIV-1 Nef-mediated increase of IL-6 and IL-8

**Figure 4:**
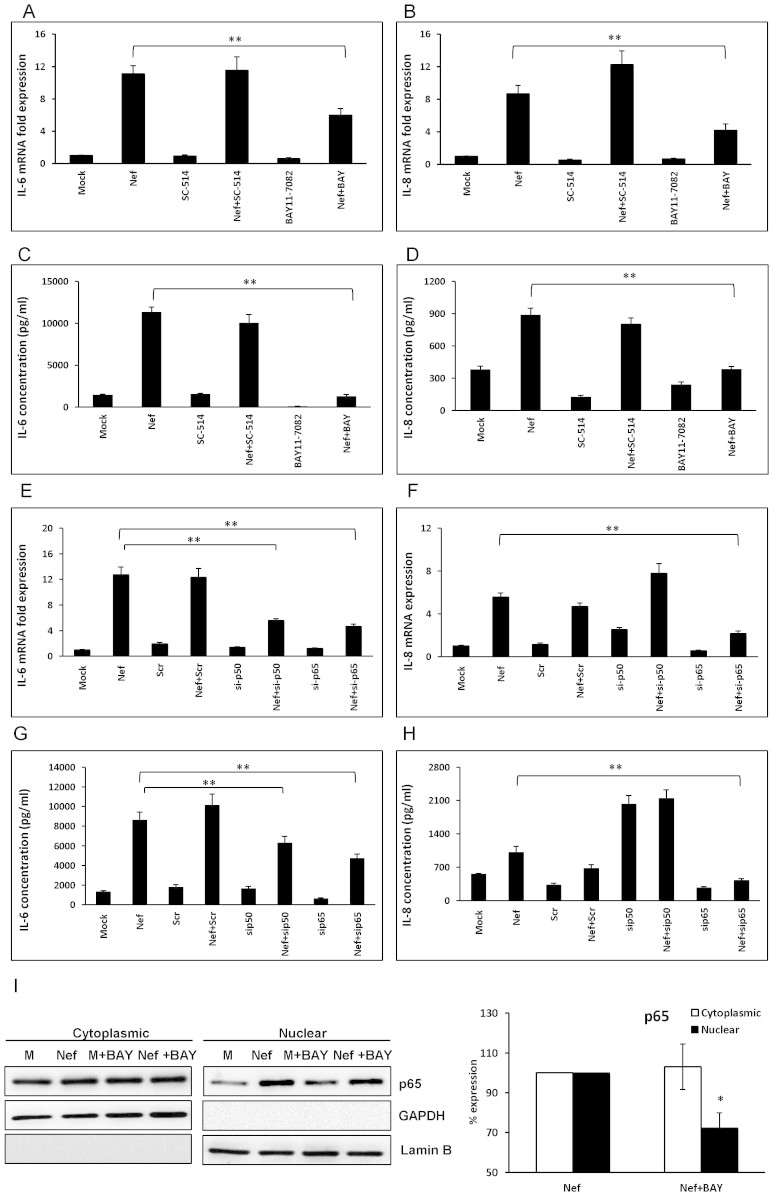
HIV-1 Nef induces IL-6 and IL-8 expression through the NF-κB pathway. SVGA cells were treated with 20 μM SC-514 or 10 μM Bay11-7082 prior to the transfection. IL-6 and IL-8 mRNA (A–B) as well as protein (C–D) levels were determined at 6 and 48 h post transfection, respectively. For knockdown of p50 and p65, the cells were transfected with the siRNA followed by transfection of nef-encoding plasmid as described in Materials and Methods. The expression levels of IL-6 and IL-8 mRNA and protein were determined at 6 h and 48 h post-transfection by real-time RT-PCR (E–F) and multiplex cytokine assay (G–H), respectively. The values presented for mRNA are relative to the mock-transfected controls. In separate experiments, SVGA cells were seeded in 6-well plates and treated with 10 μM BAY11-7082 1 h prior to transfection with a plasmid encoding HIV-1 Nef. The cells were harvested at 3 h for isolation of cytoplasmic and nuclear proteins followed by western blotting for determination of p65 expression levels in the cytosol and nucleus. The figure shows one set of data that is representative of 3 independent experiments. The percentage expression of p65 is expressed relative to the nef-transfected wells at 3 h which was considered 100% (I). GAPDH was used as a loading control for cytoplasmic extracts and Lamin B was used as a loading control for nuclear extracts. Each of the bars represents mean ± SE of three independent experiments in triplicates. Statistical analysis was performed using one way ANOVA with the LSD post-hoc test in which * represents p-value ≤ 0.05 and ** represents p-value ≤ 0.01.

The above data suggested that HIV-1 Nef induced NF-κB activation and translocation of p65. The question remains as to whether these phosphorylation events lead to IL-6 and IL-8 induction specifically by HIV-1 Nef. To investigate the involvement of IKKs in regulation of IL-8, a specific inhibitor for IKK-2, SC-514 (20 μM), was added to the culture medium 1 h prior to transfection with HIV-1 Nef-expressing plasmid. However, it failed to abrogate the increased expression of IL-6/IL-8 (Fig. 4A–D). Then, a non-selective inhibitor for IKK1 and IKK2, BAY11–7082 (10 μM) was utilized and the expression levels of IL-6 were significantly reduced by 44.5 ± 7.3% at the level of mRNA (6 h post-transfection) (Fig. 4A) and 87.4 ± 2.6% at the level of protein (48 h post-transfection) (Fig. 4C). The expression levels of IL-8 were significantly reduced by 48.3 ± 6.7% at the level of mRNA (6 h post-transfection) (Fig. 4B) and 49.7 ± 3.8% at the level of protein (48 h post-transfection) (Fig. 4D).

To evaluate the role of NF-κB subunits p50 and p65 in IL-6 and IL-8 induction by HIV-1 Nef, we used specific siRNAs to knockdown p50/p65. The siRNAs were transfected 48 h prior to transfection with the HIV-1 Nef-expressing plasmid. The siRNAs for both p50 and p65 showed significant reduction of IL-6 by 56.1 ± 2.3% and 63.2 ± 2.8% at the mRNA level, respectively (Fig. 4E). Similarly, the IL-6 protein expression levels in the cell culture supernatant were reduced by both p50 and p65 siRNAs, respectively (Fig. 4G). These results suggest that both NF-κB subunits p50 and p65 are involved in HIV-1 Nef-mediated IL-6 release in astrocytes. However, the siRNA against p50 did not abrogate the HIV-1 Nef-mediated increase of IL-8 at mRNA, rather, it up-regulated IL-8 production in HIV-1 Nef-transfected cells. On the other hand, the siRNA against p65 showed significant reduction of IL-8 by 61.2 ± 4.2% (Fig. 4F). Similarly, the IL-8 protein expression levels in the cell culture supernatant were reduced by the siRNA for p65, but not by the siRNA for p50 (Fig. 4H). These results suggest that only the p65 subunit of NF-κB is involved in HIV-1 Nef-mediated IL-8 release in astrocytes, suggesting that the p65 homodimer is essential for IL-8 induction by HIV-1 Nef.

Next, to confirm the link of IKK and NF-κB translocation, BAY11-7082 (10 μM) was added to the culture medium 1 h prior to transfection with HIV-1 Nef-expressing plasmid. Cells were harvested at 3 h after starting transfection and nuclear and cytoplasmic protein fractions were prepared and p65 levels in the cytoplasm and nucleus were determined by western blot. The nuclear expression of p65 protein induced by HIV-1 Nef was significantly abrogated by BAY11-7082 by 27.72 ± 7.5% compared with that in the HIV-1 Nef-transfected wells without inhibitors (Fig. 4I).

### IL-6 and IL-8 expression is induced by HIV-1 Nef in a PI3K-PKC dependent, Akt independent manner

**Figure 5:**
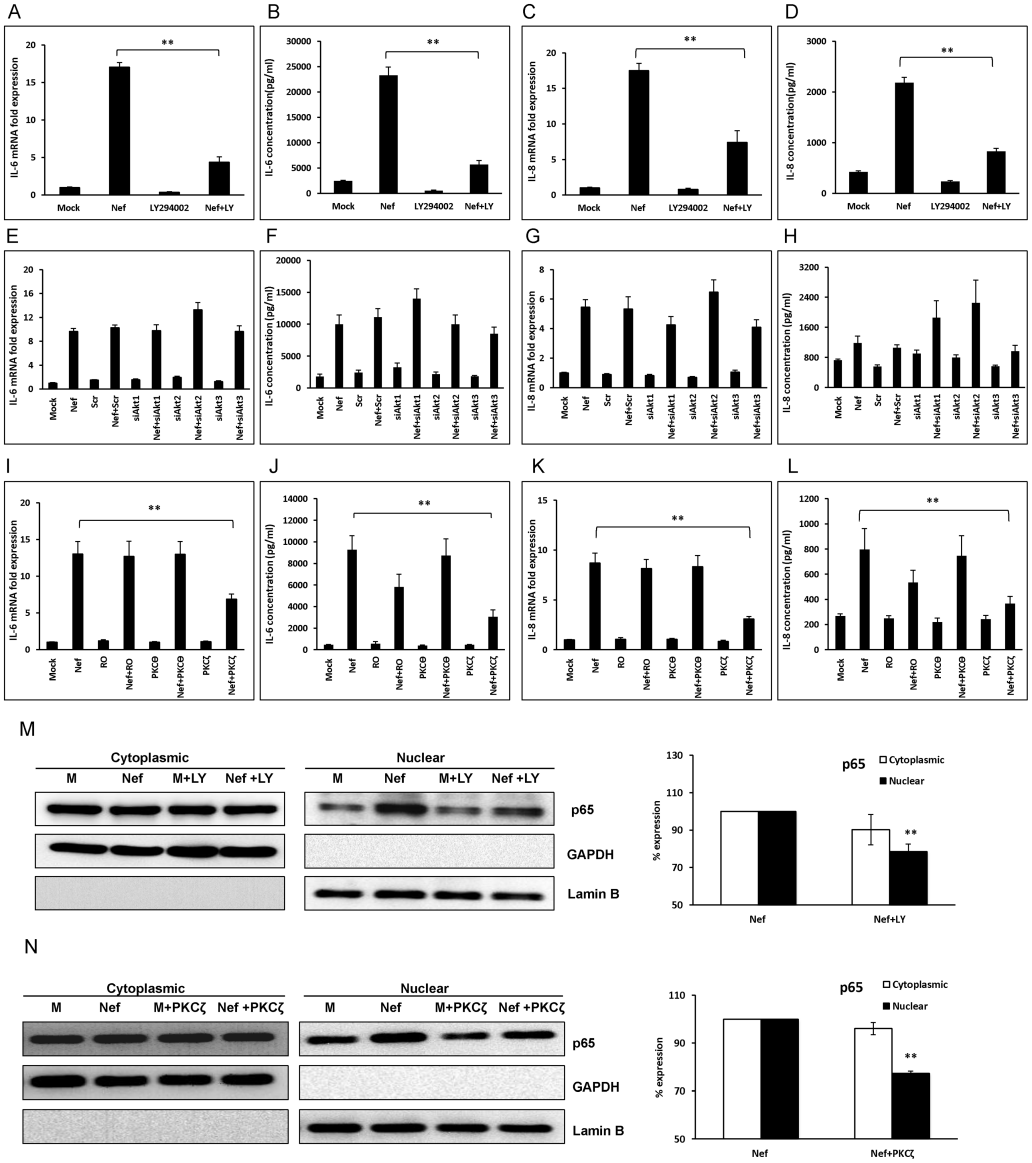
The PI3K-PKC pathway is involved in Nef-mediated IL-6 and IL-8 increases. SVGA astrocytes were pretreated with 10 μM of specific PI3K inhibitor LY294002 (A–D) or transfected with siRNA against Akt1/2/3 (E–H) prior to transfection with a nef-encoding plasmid. The expression levels of IL-6 and IL-8 mRNA and protein were determined at 6 h and 48 h post-transfection by real-time RT-PCR (A, C, E and G) and multiplex cytokine assay (B, D, F and H), respectively. In separate experiments, the cells were pretreated with a chemical inhibitor for PKCδ (Rottlerin) (3 μM), a specific pseudopeptide inhibitor for PKCθ (5 μM) or a specific pseudopeptide inhibitor for PKCζ (5 μM), prior to transfection with a nef-encoding plasmid. The expression levels of IL-6 and IL-8 were determined at 6 h and 48 h post-transfection by real-time RT-PCR (I and K) and multiplex cytokine assay (J and L), respectively. The values represented for mRNA are relative to the mock-transfected controls. In order to determine whether PKC mediates p65 translocation 7 × 10^5^ SVGA astrocytes were seeded in 6-well plates and pre-treated with LY294002 (10 μM) or pseudopeptide inhibitor for PKCζ (5 μM) 1 h prior to transfection with a plasmid encoding HIV-1 Nef (M–N). The cells were harvested at 3 h for isolation of cytoplasmic and nuclear proteins followed by western blotting for determination of p65 expression levels in the cytosol and nucleus. The figure shows one set of data that is representative of 3 independent experiments. The percentage expression of p65 was calculated relative to the nef-transfected wells at 3 h which was considered 100%. GAPDH was used as a loading control for cytoplasmic extracts and Lamin B was used as a loading control for nuclear extracts. Each of the bars represents mean ± SE of 3 independent experiments which were performed in triplicate. Statistical analysis was performed using one-way ANOVA with an LSD post-hoc test in which * represents p-value ≤ 0.05 and ** represents p-value ≤ 0.01.

In order to determine the upstream signaling pathways involved in NF-κB activation by HIV-1 Nef, we first investigated the potential role of PI3K in HIV-1 Nef-mediated IL-6 and IL-8 expression. A chemical inhibitor of PI3K, LY294002 (10 μM), was added to the culture medium 1 h prior to transfection with HIV-1 Nef-expressing plasmid. The expression levels of IL-6 were significantly reduced by 74.4 ± 4.5% at the level of mRNA (6 h post-transfection) (Fig. 5A) and 75.5 ± 3.4% at the level of protein (48 h post-transfection) (Fig. 5B). The expression levels of IL-8 were significantly reduced by 57.7 ± 10.6% at the level of mRNA (6 h post-transfection) (Fig. 5C) and 62.0 ± 2.6% at the level of protein (48 h post-transfection) (Fig. 5D).

Since Akt is a common downstream signaling molecule following PI3K activation, we then determined the involvement of different isoforms of Akt in the production of IL-6 and IL-8 induced by HIV-1 Nef. Specific siRNAs for Akt1, Akt2 and Akt3 were transfected 48 h prior to transfection with HIV-1 Nef-expressing plasmid. The specificity of these siRNAs for Akt isoforms had already been determined in previous study from our laboratory^31^. Surprisingly, none of the Akt siRNAs were able to abrogate IL-6/IL-8 expression at either mRNA or protein levels (Fig. 5E–H).

An alternative pathway which PI3K leads to is the protein kinase C (PKC) family, of which PKCδ, PKCθ and PKCζ have been shown to regulate NF-κB activity^32^,^33^,^34^. Based on that, we wanted to determine the role of different PKC isotypes in the HIV-1 Nef-mediated increase of IL-6 and IL-8. Rotterlin, an inhibitor of PKCδ (3 μM), as well as a specific pseudopeptide for PKCθ (5 μM) and a specific pseudopeptide for PKCζ (5 μM), were added to the culture medium 1 h prior to transfection. Among them, only the pseudopeptide for PKCζ showed significant reduction of IL-6 by 47.3 ± 5.2% and 67.0 ± 7.5% at the mRNA level and protein level, respectively (Fig. 5I–J). Similarly, only the pseudopeptide inhibitor for PKCζ showed significant reduction of IL-8 by 64.4 ± 2.8% and 54.0 ± 7.8% at the mRNA level and protein level, respectively (Fig. 5K–L).

To confirm the link of PI3K-PKC and NF-κB translocation, in separate experiments LY294002 or the pseudopeptide for PKCζ were added to the culture medium 1 h prior to transfection. Cells were harvested after 3 h for nuclear and cytoplasmic protein preparation and p65 levels in the cytoplasm and nucleus were determined by western blot. The nuclear expression of p65 protein induced by HIV-1 Nef was significantly abrogated by LY294002 and by the pseudopeptide for PKCζ by 21.4 ± 3.9% and 22.6 ± 0.9%, respectively, compared with that in the HIV-1 nef-transfected wells without inhibitors (Fig. 5M–N).

### The role of p38 MAPK pathway in IL-6 and IL-8 expression induced by HIV-1 Nef

**Figure 6:**
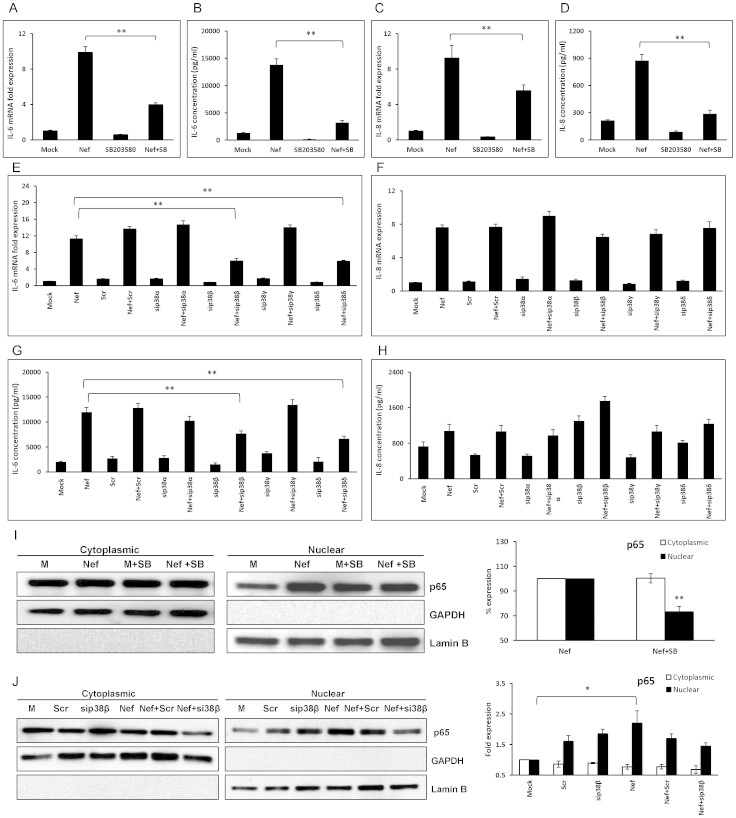
Involvement of p38 MAPK pathway in Nef-mediated IL-6 and IL-8 increase. SVGA astrocytes were pretreated with 10 μM of specific p38α and p38β inhibitor SB203580 (A–D) or transfected with siRNA against p38 isoforms (E–H) prior to transfection with nef-encoding plasmid. The expression levels of IL-6 and IL-8 mRNA and protein were determined at 6 h and 48 h post-transfection by real-time RT-PCR (A, C, E and F) and multiplex cytokine assay (B, D, G and H), respectively. In separate experiments, 7 × 10^5^ SVGA astrocytes were seeded in 6-well plates and pre-treated with SB203580 (10 μM) or transfected with siRNA against p38β prior to transfection with a plasmid encoding HIV-1 Nef (I–J). The cells were harvested at 3 h for isolation of cytoplasmic and nuclear proteins followed by western blotting for determination of p65 expression levels in the cytosol and nucleus. The figure shows one set of data that is representative of 3 independent experiments. The percentage expression of p65 was calculated relative to the nef-transfected wells at 3 h which was considered 100%. GAPDH was used as a loading control for cytoplasmic extracts and Lamin B was used as a loading control for nuclear extracts. The values represented for mRNA are relative to the mock-transfected controls. Each of the bars represents mean ± SE of three (A–D and I–J) or five (E–H) independent experiments in triplicates. Statistical analysis was performed using one-way ANOVA with an LSD post-hoc test t in which * represents p-value ≤ 0.05 and ** represents p-value ≤ 0.01.

Previous reports have indicated that the expression of IL-6 and IL-8 are regulated by p38 MAPK in astrocytes^35^,^36^. Thus, we wanted to determine the possible role of the p38MAPK pathway in HIV-1 Nef-mediated induction of IL-6 and IL-8 by using both chemical inhibitors and siRNA approaches. First, a chemical inhibitor of p38α and p38β, SB203580, was added to the culture medium at a concentration of 10 μM 1 h prior to transfection with a plasmid encoding HIV-1 Nef. The expression levels of IL-6 were decreased by 59.6 ± 2.0% and 76.7 ± 3.3% at the level of mRNA (6 h post-transfection) and protein (48 h post-transfection), respectively (Fig. 6A–B). The expression levels of IL-8 were also decreased by 39.9 ± 7.0% and 67.1 ± 5.8% at the level of mRNA (6 h post-transfection) and protein (48 h post-transfection), respectively (Fig. 6C–D).

Then, to further determine the potential involvement of different isoforms of p38 MAPK, cells were transfected with siRNAs specific for the p38α, p38β, p38γ and p38δ isoforms 48 h prior to transfection with HIV-1 Nef-expressing plasmid. The specificity of the siRNAs for p38 isoforms was determined in a previous report from our laboratory. The siRNAs for p38β and p38δ reduced the expression level of IL-6 mRNA (6 h post-transfection) by 43.7 ± 6.1% and 44.4 ± 2.1%, respectively (Fig. 6E). Similarly, both siRNAs for p38β and p38δ were able to abrogate the level of IL-6 protein at 48 h (Fig. 6F). Surprisingly, in the same sets of experiments (5 independent experiments) none of them showed a significant reduction of IL-8 at mRNA or protein level (Fig. 6G–H).

To confirm that blockage of p38 could inhibit NF-κB translocation, cells were either pre-treated with SB203580 (10 μM) or transfected with siRNA for p38β (50 nM) prior to transfection with HIV-1 Nef-expressing plasmid. Nuclear and cytoplasmic proteins were obtained from the cells at 3 h after transfection and p65 levels in the cytoplasmic and nuclear fractions were determined by western blot. SB203580 significantly reduced the nuclear expression level of p65 in the HIV-1 Nef-transfected cells by 26.8 ± 4.2% compared to that in the cells without the inhibitor (Fig. 6I). The p65 nuclear expression level in the cells transfected with both p38β siRNA and HIV-1 Nef-expressing plasmid showed 34% reduction compared to that in the cells only transfected with HIV-1 Nef-expressing plasmid (Fig. 6J). The data suggest that HIV-1 Nef regulates the expression of IL-6 through p38β and p38δ isoforms, of which p38β causes activation of NF-κB.

### The role of transcription factors C/EBP and AP-1 in IL-6 expression induced by HIV-1 Nef

**Figure 7:**
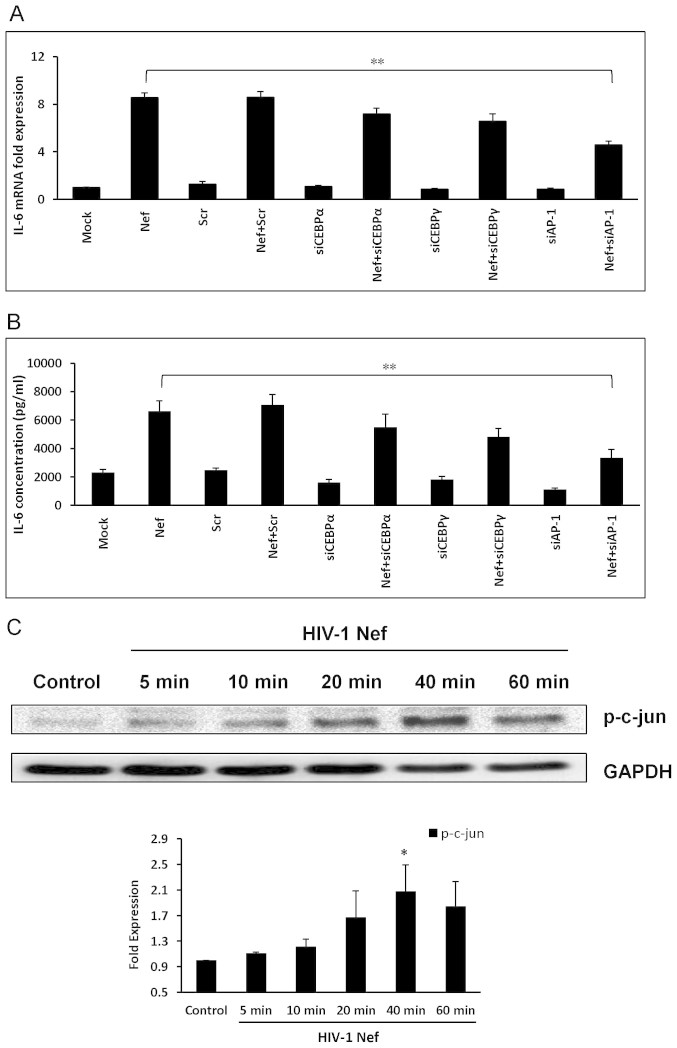
Involvement of transcriptional factors C/EBP and AP-1 in Nef-mediated increase of IL-6 expression. For knockdown of C/EBPα, C/EBPγ and AP-1, the cells were transfected with the siRNA followed by transfection of nef-encoding plasmid as described in Materials and Methods. The expression levels of IL-6 and IL-8 mRNA and protein were determined at 6 h and 48 h post-transfection by real-time RT-PCR (A) and multiplex cytokine assay (B), respectively. The values represented for mRNA are relative to the mock-transfected controls. Each of the bars represents mean ± SE of three independent experiments which were performed in triplicate. In separate experiments, 7 × 10^5^ SVGA astrocytes were seeded in 6-well plates and were treated with recombinant HIV-1 Nef at a concentration of 20 nM. The cells were harvested at the indicated times for preparation of whole cell lysates following by western blotting for determination of p-c-jun levels. The figure shows one set of data that is representative of 3 independent experiments. Levels of p-c-jun were quantified by spot densitometry and normalized to GAPDH. Levels of p-c-jun are presented as mean ± SE relative to control and represent data obtained from 3 independent experiments (C). Statistical analysis was performed using one-way ANOVA with an LSD post-hoc test t in which * represents p-value ≤ 0.05 and ** represents p-value ≤ 0.01.

It has been demonstrated that transcription factors like C/EBPα, C/EBPγ and AP-1 can be activated by p38δ signaling, and HIV-1 Nef has been demonstrated to activate AP-1 in U937 monocytic cells^37^. Thus, the involvement of those transcription factors was also determined in the regulation of IL-6 by HIV-1 Nef. Cells were transfected with specific siRNAs against C/EBPα, C/EBPγ and AP-1 48 h prior to transfection with HIV-1 Nef-expressing plasmid. IL-6 expression was significantly abrogated by AP-1 siRNA by 46.3 ± 3.6% and 41.8 ± 9.4% at the level of mRNA (6 h post-transfection) and protein (48 h post-transfection), respectively (Fig. 7A–B). The results above imply that AP-1 plays an important role in the induction of IL-6 by HIV-1 Nef. AP-1 as a functional transcription factor is comprised of a homodimer/heterodimer of the Jun family or a heterodimer of both Fos and Jun, most commonly c-Fos and c-jun^38^. So we investigated whether the c-jun subunit can be phosphorylated by HIV-1 Nef. The levels of phosphorylation of c-jun in whole cell lysates of SVGA treated with HIV-1 recombinant Nef protein from 5 min to 60 min were measured by western blot. The phosphorylation level of c-jun showed significant increase (∼ 2 fold) in HIV-1 Nef-treated cells compared with that in untreated controls at 40 min after HIV-1 Nef exposure (Fig. 7C).

## Discussion

HIV-1 Nef plays an essential role in the maintenance of high viral load and disease progression during HIV infection^39^. The functions of HIV-1 Nef have been established mainly in T lymphocytes, including down-regulation of cell surface marker, enhancement of viral infection, and activation of cellular signaling^40^. In addition, HIV-1 Nef resulted in neuronal cell death, possibly through the cytotoxicity of IP-10 produced by astrocytes^41^. Other mechanisms responsible for HIV-1 Nef-induced neurotoxicity include disruption of BBB, production of proinflammatory cytokines and increased levels of oxidative stress^14^,^15^,^42^.

IL-6, a pro-inflammatory cytokine, is an activator of acute phase responses and elevated levels of IL-6 were found in the CSF of HIV-infected patients with dementia, indicating that IL-6 may be involved in the pathogenesis of HAND^22^,^23^,^24^. IL-8, identified as one of the first members of chemokine family, has been demonstrated to enhance HIV replication in macrophages, microglia and T cells^43^,^44^. Apart from that, elevated levels of IL-8 were found in the CSF of HIV-infected patients with dementia compared with than in HIV-1 infected individuals without neurological impairment, indicating that IL-8 may be involved in the pathogenesis of HAD^29^.

In the present study, when the primary human fetal astrocytes were exposed to exogenous HIV-1 Nef, there was significant IL-6 and IL-8 induction at mRNA and protein levels, demonstrating a direct association between HIV-1 Nef and increased IL-6 and IL-8. Notably, the average concentration of IL-8 obtained from the supernatant of human fetal astrocytes treated with HIV-1 Nef was ∼ 4 ng/ml, which is much higher than the concentration found in the CSF of HAD patients (140 pg/ml)^32^. To elucidate the mechanisms responsible for HIV-1 Nef-mediated IL-6 and IL-8 induction, we endogenously expressed HIV-1 Nef in SVGA astrocytic cell line with a plasmid encoding HIV-1 Nef. Similar to the results seen with the primary astrocytes, there was a significant increase of IL-6 and IL-8 at both mRNA levels and protein levels.

Previous studies have identified an NF-κB binding site in the IL-6 promoter^45^. Our study demonstrated that HIV-1 Nef caused increased phosphorylation of IKK and IκBα followed by activation and nuclear translocation of p65 in SVGA cells. Also, both the siRNA for p65 and p50 were able to abrogate the IL-6 levels, suggesting that both the subunits are important for transcriptional control of IL-6. In contrast to our results, a previous study showed that the activation of the p50/p50 homodimer was responsible for IL-6 production in monocyte-derived macrophages treated with HIV-1 Nef^46^. The difference between our results and the previous report may be due to differences between astrocytes and macrophages. As for the regulation of IL-8, only siRNA against p65 significantly abrogated IL-8 expression at the mRNA and protein levels, while siRNA against p50 showed the opposite effect with increased IL-8. Our results demonstrated that the p65/p65 homodimer is mainly responsible for HIV-1 Nef-induced IL-8 expression. Importantly, this conclusion is supported by other studies showing an NF-κB binding site with a high affinity for p65 homodimers but not for p65/p50 heterodimers or p50 homodimers in the IL-8 promoter^47^,^48^. In addition, we determined the individual involvement of IKK1 and IKK2 isozymes in the HIV-1 Nef-mediated IL-6 and IL-8 expression by using SC-514, an IKK2 specific inhibitor, and BAY1170-82, a non-selective inhibitor for IKK1 and IKK2. The data demonstrated that BAY1170-82, but not SC-514, significantly reduced the expression level of IL-6 and/or IL-8 in HIV-1 nef-transfected cells, implying that IKK2 is not essential for induction of IL-6/IL-8 by HIV-1 Nef.

HIV-1 Nef can interact with PI3K via direct binding to the regulatory p85α subunit of class Ia PI3K^49^. Our results showed that LY294002, an inhibitor of PI3K activity, significantly abrogated the HIV-1 Nef-induced increase of IL-6 and IL-8 as well as the nuclear expression of p65. To investigate the role of downstream signaling molecules targeted by PI3K, we first performed siRNA transfections against three Akt isoforms. However, none of the siRNAs had any inhibitory effect on IL-6/IL-8. This suggested that an alternative pathway may be responsible for HIV-1 Nef-mediated IL-6 and/or IL-8 induction. Previously, PKC had been shown to be a link between PI3K and NF-κB in various cell types, and among all the isoforms it was reported that PKCδ, PKCθ and PKCζ are activators of NF-κB^32^,^33^,^34^. To elucidate the role of the PKC isoforms, we utilized three different inhibitors for PKCδ, PKCθ and PKCζ, of which only the PKCζ pseudopeptide inhibitor resulted in a substantial reduction of IL-6 and IL-8 in cells transfected with the HIV-1 Nef-expressing plasmid. Moreover, both LY294002 and PKCζ inhibitor could abrogate the HIV-1 Nef-mediated translocation of p65. Taken together, these results lead to the conclusion that IL-6 and IL-8 expression is induced by HIV-1 Nef in a PI3K-PKCζ-NF-κB dependent, Akt-independent manner.

The MAPKs are a group of serine/threonine-specific kinases that are activated in response to a variety of extracellular stimuli, and five distinct kinases have been identified: ERK1/2, JNKs, p38 (p38α, p38β, p38γ and p38δ), ERK3/4, and ERK5^50^. Among them, the phosphorylation of p38 kinase can lead to the activation of downstream transcription factors like NF-κB, p53, C/EBPβ, C/EBPα, C/EBPγ and AP-1^51^. In our study, SB203580, an inhibitor for only p38α and p38β was first utilized, and this resulted in a reduction of IL-6 and IL-8 at both mRNA and protein levels. Then, to confirm the individual role of each of the p38 isoforms, specific siRNAs for p38α, p38β, p38γ and p38δ were used and the results revealed that p38β and p38δ were involved in the HIV-1 Nef-mediated IL-6 increase. Furthermore, by using either SB203580 or siRNA against p38β, there was abrogation of HIV-1 Nef-induced increase of p65 translocation into the nucleus, indicating that p38β is another upstream signaling specifically activated by HIV-1 Nef which leads to p65 translocation. On the other hand, none of these siRNAs acted to reduce IL-8 expression levels. The discrepancy between the results of IL-8 inhibition levels by chemical inhibitor and siRNA knockdown for p38 might be due to several reasons. First, SB203580 resulted in down-regulation of IL-8 in mock-transfected cells, implying that there was inhibition of basal levels of IL-8 mRNA and protein in astrocytes. Thus, if the fold expression of IL-8 in HIV-1 nef-transfected cells with SB203580 treatment is normalized with that in mock-transfected cells with SB203580 treatment, it is comparable to IL-8 expression of HIV-1 nef-transfected cells. Also, SB203580 may have a non-specific effect other than inhibiting p38α and p38β based on previous reports showing that SB203580 could cause inhibition of PDK1 and Akt, or activation of other pathways like JNK and ERK^52^,^53^.

In addition to NF-κB, transcription binding sites AP-1 and C/EBP also exist on IL-6 promoter, and mutations in the putative binding sites for AP-1 and C/EBPα have been shown to reduce IL-6 activation^54^. Therefore, we wanted to explore the role of C/EBPα, C/EBPγ and AP-1, which are downstream targets of p38δ. First, siRNAs against C/EBPα, C/EBPγ and AP-1 were utilized and the results showed that siRNA for AP-1 significantly reduced IL-6 at both mRNA and protein levels. To confirm that AP-1 subunit c-jun is activated by HIV-1 Nef, astrocytes were treated with HIV-1 Nef protein and increased phosphorylation levels of c-jun were seen in HIV-1 Nef-treated cells and the peak level was found to be at 40 min. Taken together, we can conclude that NF-κB and AP-1 are the major transcription factors responsible for HIV-1 Nef-mediated IL-6 induction. We also tested IL-8 following transfection of those siRNAs, but these siRNAs did not affect HIV-1 Nef-mediated IL-8 expression (data not shown). Reports have shown that although the IL-8 promoter contains binding sites for AP-1 and C/EBP along with NF-κB, activation of NF-κB controls the regulation of IL-8^55^.

**Figure 8:**
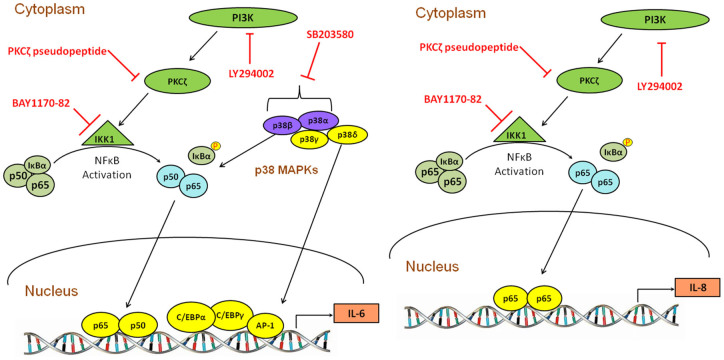
Schematic of the signaling pathways involved in IL-6 and IL-8 up-regulation caused by HIV-1 Nef in astrocytes: HIV-1 Nef activates IL-6 and IL-8 expression utilizing different pathways. The molecules whose involvement were determined using inhibitors were depicted in green color, and the molecules whose involvement were determined using siRNAs were depicted in yellow color, while purple color indicated that both inhibitors and siRNAs were used.

To date other HIV-1 proteins namely HIV-1 gp120 and HIV-1-Tat has been mostly implicated in neuroinflammation as these proteins have been shown to cause overexpression of variety of proinflammatory cytokines including IL-1β^56^, IL-6^56^,^57^,^58^,^59^,^60^, MCP-1^60^,^61^,^62^, IL-8^57^,^61^,^63^ and CCL5^58^,^64^ among others. More recently HIV-1 Nef has also been found to play a possible role in neuroinflammation as evident by increased expression of MCP-1^15^ and CCL5^31^. Therefore, we undertook a detailed study to determine whether and how HIV-1 nef causes overexpression of IL-6 and IL-8 in astrocytes. In summary, in our study the contribution of different transcription factors was characterized in HIV-1 Nef-mediated cytokine production and the data suggested NF-κB p50 and p65 as well as AP-1 are involved in HIV-1 Nef-induced IL-6, while only p65 homodimers are involved in HIV-1 Nef-induction of IL-8. Furthermore, different upstream signaling pathways such as PI3K-PKCζ and p38β and p38δ MAPK were also stimulated by HIV-1 Nef which differentially regulate the expression levels of IL-6 and IL-8. The involvement of those pathways is depicted in Fig. 8. Based on that, possible therapeutic inhibitors and siRNAs targeted against those signaling molecules can be developed for treatment of neuroinflammation and the cognitive deficits seen in HIV HAND patients.

## Methods

### Cell culture and treatment

The SVGA astrocytic cell line was a generous gift from Dr. Avi Nath^65^. The cells were maintained in Dulbecco's Modified Eagle Medium (DMEM) containing 10% FBS and 1% gentamicin at 37°C in a 5% CO_2_ environment. SVGA cells were cultured in 20 ml media in a 150 mm^2^ flask seeded with an initial amount of 3.2 × 10^6^ cells. SVGA cells were passaged every other day and the cells were allowed to adhere overnight. Two to three passages were maintained before the SVGA cells were used for experiments.

The primary fetal astrocytes were isolated from brains obtained from fetuses aborted between 80 to 110 days. This material was obtained from the Birth Defects Research Lab (BDRL) (Seattle, WA). Six donors were used in experiments: Donor 1_24942, Donor 2_25528, Donor 3_25175, Donor 4_25177, Donor 5_26229 and Donor 6_26239.

All transfections and treatments were performed in 6-well or 12-well plates. Transfections of SVGA were performed using Lipofectamine™ 2000 as per the manufacturer's instructions (Invitrogen Inc., Carlsbad, CA) with some modifications as published in our earlier reports^63^,^64^,^66^. After 5 h, transfection medium was replaced by fresh DMEM containing 10% FBS. Cells were then harvested at the indicated times for RNA or protein isolation. SC-514 (IKK 2 inhibitor), Bay11-7082 (IKK inhibitor), LY294002 (PI3K inhibitor), Rottlerin (PKCδ inhibitor), SB203580 (p38 MAPK inhibitor) (Cayman Chemicals, Ann Arbor, MI), PKCθ pseudopeptide (Santa Cruz Biotechnology, Santa Cruz, CA) or PKCζ pseudopeptide (Sigma-Aldrich, St. Louis, MO) were added 1 h prior to transfection with HIV-1 nef plasmid. The nef plasmid was obtained through the NIH AIDS Reagent Program, Division of AIDS, NIAID, NIH: p96ZM651nef-opt from Drs. Yingying Li, Feng Gao, and Beatrice H. Hahn^67^. Alternatively, SVGA cells or primary astrocytes were treated with recombinant HIV-1 Nef Protein. The recombinant HIV-1 Nef was added into culture medium at a concentration of 20 nM (MW = 26.5 KDa) and the cells were harvested at the indicated time for RNA or protein isolation. The recombinant HIV-1 Nef Protein was obtained from the NIH AIDS Reagent Program, Division of AIDS, NIAID, NIH: HIV-1 Nef Protein.

### Real time RT-PCR and multiplex cytokine assay

Total RNA was extracted using RNeasy mini kits (Qiagen, Valencia, CA) as described in the manufacturer's protocol. Real time reverse transcriptase polymerase chain reactions (RT-PCR) using Bio-Rad iCycler was performed to determine the mRNA expression levels. The reaction conditions included reverse transcription at 50°C for 30 min, denaturation at 95°C for 15 min, followed by amplification of IL-6 for 50 cycles (95°C for 15 sec and 57.5°C for 1 min) or amplification of IL-8 for 55 cycles (95°C for 30 sec and 61°C for 30 sec). The primers and probes were obtained from Integrated DNA Technology (Coralville, IA). The forward primer (5′ GGT ACA TCC TCG ACG GCA TC 3′), reverse primer (5′ CCA GTG CCT CTT TGC TGC TT 3′), and probe (5′ FAM CAG CCC TGA GAA AGG AGA CAT GTA ACA GGA AA-3′ BHQ) were used for amplification of IL-6; the forward primer (5′ CTC TTG GCA GCC TTC CTG ATT 3′), reverse primer (5′ TAT GCA CTG ACA TCT AAG TTC TTT AGC A 3′), and probe (5′ FAM-CTT GGC AAA ACT GCA CCT TCA CAC AGA-3′ BHQ) were used for amplification of IL-8. Hypoxanthine-guanine phosphoribosyl-transferase (HPRT) was used as a house-keeping gene to normalize gene expression (forward primer 5′ GCT TTC CTT GGT CAG GCA GTA 3′ and reverse primer 5′ CCA ACA CTT CGT GGR GTC CTT T 3′). The data was analyzed using the 2^−ΔΔCT^ method as used in our previous study^63^.

Cell culture supernatants were collected from each experimental well at the indicated time followed by centrifugation at 3,000 rpm for 10 min at 4°C to remove floating cells. Cytokine protein concentrations were analyzed by a multi-cytokine bead assay system (Bio-Plex ProTM Human Cytokine Group I 3-Plex assay). Assays were performed according to the manufacturer's instructions (Bio-Rad, Hercules, CA).

### Transfection with siRNAs

NF-κB, and p38 α/β/γ/δ siRNAs, as well as siRNAs against C/EBPα, C/EBPγ, AP-1, and Akt (1/2/3) (Life Technologies, Grand Island, NY) were transfected 48 h prior to nef transfection. 6 × 10^5^ cells in each well were transfected with 50 nM siRNA in serum-free media in 6-well plates. After 24 h, the transfection medium was replaced with fresh DMEM supplemented with 10% FBS. Cells were then trypsinised 8 h later and re-seeded at a density of 2.5 × 10^5^ per well in 12-well plates. Nef transfection was then performed the next day as described above and cells were harvested at different time points for determination of cytokines at both RNA and protein levels. Transfection with 50 nM Silencer Select negative control siRNA (Ambion, Austin, TX) was used as the scrambled control.

### Immunocytochemistry

SVGA cells were seeded on 1.5mm cover slips in 6-well plates followed by either mock transfection, or transfection with a plasmid encoding HIV-1 Nef. After 5 h, transfection medium was replaced by fresh DMEM containing 10% FBS. Addition of 3 µM GolgiStop™ (BD Biosciences, San Jose, CA) was performed 3 h prior to harvest in order to prevent the release of IL-6 or IL-8. 6 h later, the cells were fixed with an ice-cold solution of methanol and acetone (1:1) for 20 min at 20°C. Then the cells were washed 3 times with PBS followed by blocking with 1% BSA in PBS with 0.1% Triton for 30 min. After blocking, the cells were incubated with a cocktail of rabbit anti-IL-6/anti-IL-8 antibody (1:200) (Santa Cruz Biotechnology, Santa Cruz, CA) and mouse anti-GFAP antibody (1:1500) (Abcam, Cambridge, MA) overnight in a humidified chamber. After washing 3 times with PBS with 0.1% Triton, the cells were incubated in the dark for 1 h with an anti-mouse antibody conjugated with Alexa Fluor 555 (1:1500) and an anti-rabbit antibody conjugated with Alexa Fluor 488 (1:1500) (Cell Signaling, Danvers, MA). Finally, the coverslip was transferred onto a slide with 10 µl of Vectashield mounting medium with DAPI (Vector Laboratories, Burlingame, CA). The fluorescence microscopy was performed using a Leica TCS SP5 II Laser Scanning Confocal microscope. The images were captured using a 40× zoom lens. The intensity of IL-6 and IL-8 were calculated using imageJ software and GFAP was used as a housekeeping gene to normalize the expression levels of these cytokines.

### Western blotting

Total cell lysates were prepared using RIPA buffer (Boston BioProducts, Ashland, MA), containing 1× protease inhibitor cocktail (Thermo Scientific, Rockford, IL). In separate experiments, the cells were harvested at given time points and nuclear and cytoplasmic extracts were prepared using cytoplasmic buffer (10 mM HEPES, 50 mM NaCl, 0.5 M sucrose, 0.1 mM EDTA and 0.5% Triton × 100) and nuclear buffer (10 mM HEPES, 500 mM NaCl, 0.1 mM EDTA, 0.1 mM EGTA and 0.1% IGPAL). 20 μg of protein was loaded on a 10% acrylamide gel and electrophoresed at 90 V for approximately 3 h and transferred to a PVDF membrane at 350 mA for 90 min using Mini-PROTEAN Tetra Cell (Bio-Rad, Hercules, CA). Transferred blots were blocked in 5% nonfat dry milk for 1 h followed by overnight incubation with primary antibody (between 1–1000 to 1:2000 dilution) and 2 h incubation with a corresponding HRP-conjugated secondary antibody (1:2000 dilution). 5 washes of PBS with 0.05% Tween 20 were performed both after incubation with primary and secondary to eliminate the non-specific binding of antibodies. Proteins were visualized using the Alpha Innotech FluorChem HD2 gel documentation system (Proteinsimple, Santa Clara, CA) by adding BM chemiluminescence western blotting substrate (POD) (Roche Applied Sciences, Indianapolis, IN). Quantification was performed by spot densitometry using AlphaEase FC StandAlone software (version 6.0.0.14; Alpha Innotech, San Leandro, CA). The mouse monoclonal antibody against p−IκBα (39A1431), goat polyclonal antibody against Lamin B (C-20) and the horseradish peroxidase (HRP)-conjugated secondary antibodies (anti-goat and anti-mouse) were obtained from Santa Cruz Biotechnology (Santa Cruz, CA). The rabbit monoclonal antibodies against NF-κB p65 (D14E12), GAPDH (14C10), p-IKK-alpha/IKK-beta (C84E11), IKK-alpha (3G12), p-c-Jun (D47G9) and the horseradish peroxidase (HRP)-conjugated secondary anti-rabbit antibody were obtained from Cell Signaling Technology (Danvers, MA).

### Statistical analysis

A one way ANOVA with a least significant difference (LSD) post-hoc test was used to calculate the statistical significance in all the experiments. The results showed mean ± standard error (SE) of at least three individual experiments. P-value of ≤ 0.05 was considered to be statistically significant.

## Supplementary Material

Supplementary Information
