# Supplementary material for: Differential signaling mechanism for HIV-1 Nef-mediated production of IL-6 and IL-8 in human astrocytes
Source: Sci Rep. 2015 Jun 15;5:9867. doi: 10.1038/srep09867 (PMC4467202; doi:10.1038/srep09867)
Supplement: Supplementary Information [file srep09867-s1.pdf]

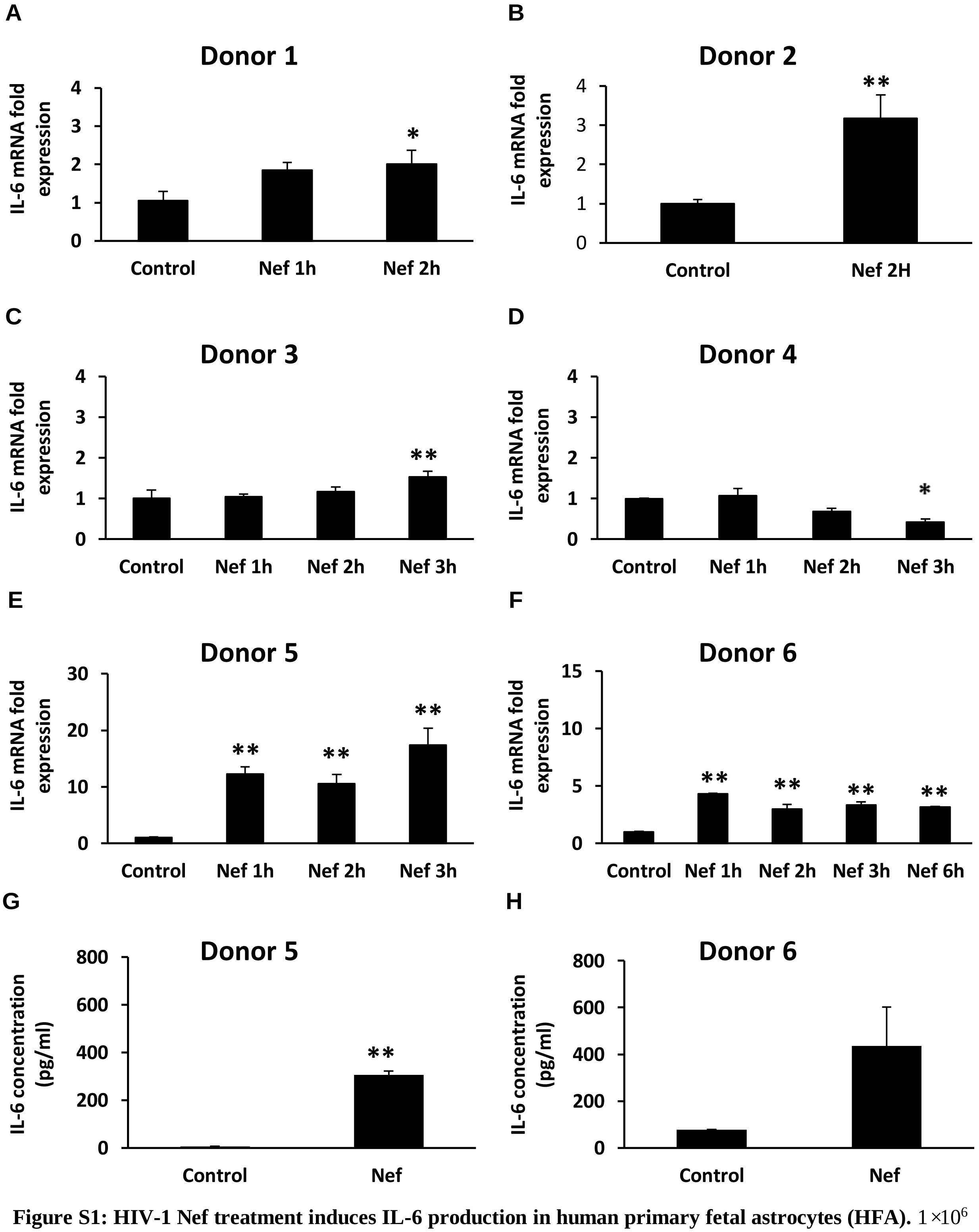

Figure S1: HIV-1 Nef treatment induces IL-6 production in human primary fetal astrocytes (HFA). 1×10^6 HFA were seeded in 12-well plates and were treated with HIV-1 Nef for the times indicated. The cells were harvested at 1 h ~ 6 h for total RNA isolation and the expression levels of IL-6 mRNA were determined by real time RT-PCR (A, B, C, D, E and F). In separate experiments, IL-6 concentrations released in the supernatants were measured at 24 h by multiplex cytokine assay (G and H). Each of the bars represents mean ± SE of triplicates. Statistical analysis was performed using one-way ANOVA with an LSD post-hoc test in which * represents p-value ≤0.05 and ** represents p-value ≤ 0.01.

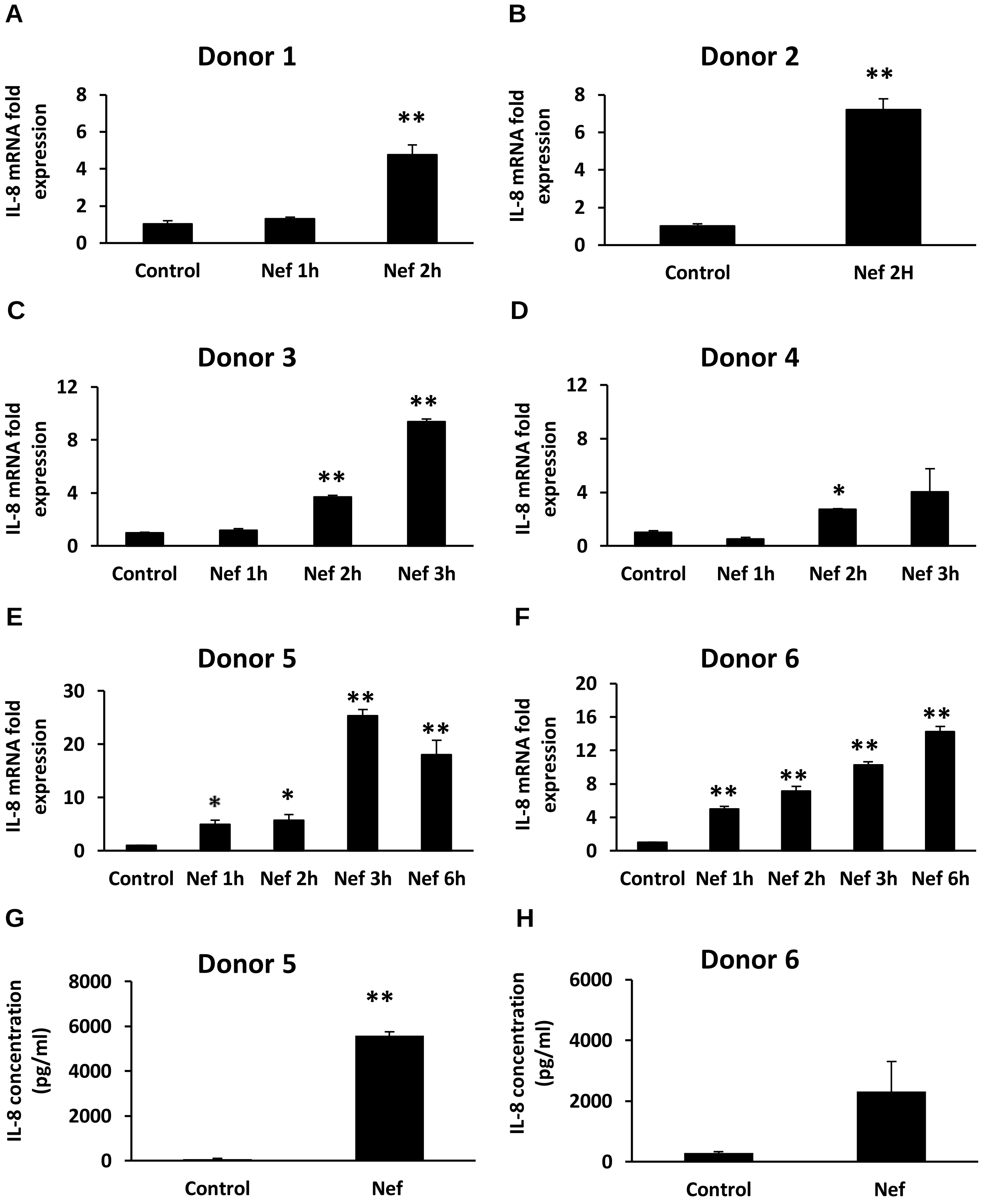

Figure S2: HIV-1 Nef treatment induces IL-8 production in human primary fetal astrocytes (HFA). 1×10^6 HFA were seeded in 12-well plates and were treated with HIV-1 Nef for the times indicated. The cells were harvested at 1 h ~ 6 h for total RNA isolation and the expression levels of IL-8 mRNA were determined by real time RT-PCR (A, B, C, D, E and F). In separate experiments, IL-8 concentrations released in the supernatants were measured at 24 h by multiplex cytokine assay (G and H). Each of the bars represents mean ± SE of triplicates. Statistical analysis was performed using one-way ANOVA with an LSD post-hoc test in which * represents p-value $\leqslant$0.05 and ** represents p-value $\leqslant$ 0.01.
